# Prevalence and Associated Factors of Depressive Disorder After Exposed Prolonged Traumatic Event

**DOI:** 10.1192/bjo.2024.187

**Published:** 2024-08-01

**Authors:** Moon-Doo Kim

**Affiliations:** Jeju National University Hospital, Jeju, Korea, Republic of

## Abstract

**Aims:**

Depressive disorder is one of the most typical psychiatric disorders that occurs after a traumatic event. However, there has been minimal research regarding the prevalence and associated factors of depression after a traumatic event. Therefore, this study aims to investigate the prevalence of depressive symptoms and associated factors in the residents of the Gangjeong village, who have been exposed to a traumatic event recently for a prolonged period.

**Methods:**

The subjects of this study were the residents of the Gangjeong village, who have been exposed to a traumatic event related to the construction of the Jeju Civilian-Military Complex Port. The questionnaires were used to assess the participants' general characteristics (sex, age, marital status, occupation, self-perceived health, etc.); in addition, for the clinical evaluation, overall stress was assessed through the Global Assessment of Recent Stress Scale (GARS), social support through Functional Social Support Questionnaire (FSSQ) and suicide risk through Mini-International Neuropsychiatric Interview-Plus (M.I.N.I-Plus). In order to evaluate the depressive symptoms, CES-D (Center for Epidemiologic Studies Depression Scale) was used.

**Results:**

In 713 subjects, the prevalence of depressive symptoms was 18.5% (95% CI=15.66–21.36) (Table 1). Multivariate logistic regression analysis identified the length of residence and marital status as factors associated with depressive symptoms (Table 2). Furthermore, the depression group has a significantly higher score of overall stress (GARS), suicide risk and the lack of social support (FSSQ), in comparison with the non-depression group (depression gr. vs non-depression gr. : 28.8 ± 15.0 vs 12.8 ± 10.1, 4.9 ± 8.0 vs 1.1 ± 3.6, 44.8 ± 13.2 vs 34.0 ± 13.9, respectively).

**Conclusion:**

The prevalence of depressive symptoms was higher among the study population compared with the general population. People exposed to the traumatic event, especially after prolonged exposure, should be assessed for environment factors, the status of overall stress, social support and the suicidal risk.

